# Predictors of optimal breastfeeding practices in Worabe town, Silte zone, South Ethiopia

**DOI:** 10.1371/journal.pone.0232316

**Published:** 2020-04-30

**Authors:** Nefsu Awoke, Tiwabwork Tekalign, Tesfanesh Lemma

**Affiliations:** 1 Department of Nursing, College of Health Science and Medicine, Wolaita Sodo University, Sodo, Ethiopia; 2 Department of Midwifery, College of Health Science and Medicine, Wolaita Sodo University, Sodo, Ethiopia; Kilimanjaro Christian Medical University College, UNITED REPUBLIC OF TANZANIA

## Abstract

**Background:**

Studies in sub-Saharan Africa indicated the overall prevalence of optimal breast feeding ranged between a lowest of 17.63% in East Africa and a highest of 46.37% in West Africa. It’s estimated that 823,000 deaths of children could be prevented every year through optimal breastfeeding practices. However optimal breastfeeding practices is low in most setting of Ethiopia. Therefore, this study aimed to assess optimal breastfeeding practices and associated factors in Worabe town.

**Method:**

A community-based analytical cross-sectional study was conducted from April 15^th^–25^th^, 2018. A systematic sampling technique was applied on 347 sampled mothers who had children greater than or equal to 2 years old. The data was entered into EpiData (version 3.1) and subsequently exported to SPSS Statistics (version 22) for analysis. Descriptive statistics were used for presenting summary data using tables and graph. Bivariate and multi variable logistic regression analysis to identify were used to identify associated factors. The statistical significance was declared at *P*<0.05.

**Result:**

Optimal breastfeeding was exhibited by 42.1% of mothers. Government employees (AOR = 8.0; 95% CI: 1.7, 36.4), families with a household income of 1,500–3,000 Ethiopian birr (AOR = 4.6; 95% CI: 1.0, 20.1), individuals knowledgeable about optimal breastfeeding practices (AOR: 5.5 95% CI: 1.6, 18.1), individuals counselled about breastfeeding practices during postnatal follow-ups (AOR = 4.940, 95% CI: 1.313, 10.195), and individuals that had a caesarean section delivery (AOR = 4.2, 95% CI: 1.2, 14.1) had a higher chance of practicing optimal breastfeeding. However, mothers who did not attend or have access to antenatal care follow-ups (AOR = 0.1, 95% CI: 0.04, 0.5) were less likely to practice optimal breastfeeding.

**Conclusions:**

Less than half of mothers breastfed their children optimally. Factors that influenced this included knowledge of optimal breastfeeding practices, total household income, the woman's occupation, access to breastfeeding counselling during postnatal care follow-ups, access to antenatal care follow-ups, and mode of delivery. It is strongly recommended that optimal breastfeeding awareness programs through health education be done in collaboration with health extension workers, and zonal health offices.

## Introduction

Optimal breastfeeding practices include the initiation of exclusive breastfeeding from the first hour of birth until 6 months of age, and then the introduction of complementary foods alongside continued breastfeeding up to 2 years of age or beyond [1]. It is considered that the period from birth to two years of age is a “critical window” of opportunity for the promotion of optimal growth, health and behavioural development of children [2]

Early initiation of breastfeeding is important for both the mother and the child. The first breast milk contains colostrum, which is highly nutritious and has antibodies that protect the newborn from diseases [3] and protecting mothers against ovarian and breast cancer deaths [4]. Optimal breastfeeding is recognized as the most cost-effective preventive measure to reduce Acute Respiratory Infection and diarrhea deaths by 50–95%, mother to child transmission of HIV by 10–20% and increase intelligence and readiness to learn [4, 5].

Worldwide, about 40.7% of infants aged 0–6 months were exclusively breastfed and 45.1% continue to be breastfed up until the age of 2. In addition, 2 in 5 new-borns wait more than one hour to initiate breastfeeding [4, 6]. In 2016, a Lancet series estimated that 823,000 deaths of children could be prevented every year through optimal breastfeeding practices [4].

Meta-analysis conducted in 29 Sub-Saharan African countries from 2010–2015 indicated the overall prevalence of optimal breast feeding ranged between a lowest of 17.63% in East Africa and a highest of 46.37% in West Africa [7]. Only about one in three African babies under six months is exclusively breastfed, due to lack of understanding of optimal feeding practices [8].

In Ethiopia according to EDHS report overall, 58% of children under age 6 months are exclusively breastfed and 67% children under age 24 months are receiving age appropriate breastfeeding (Children age 0–5 months who are exclusively breastfed + children age 6–23 months who receive breast milk and complementary foods) but only 7% of children in Ethiopia age 6–23 months meet the minimum standards with respect to all three IYCF practices (breastfeeding status, number of food groups, and times they were fed during the day or night before the survey) [3]. Similarly in South Nations and Nationalities Peoples Region (SNNPR) only 2.5% of infant and young children meet minimum dietary diversity and only 2.3% of them had the minimum acceptable diet according to EDHS 2011 [9].

Among factors identified to influence optimal breastfeeding practice were age of the child, receiving advice on breast feeding, pregnancy intention, place of residence and level of husband education [10], Maternal age, level of education [11] delivery at the health facility, normal delivery those with 2 or more than 3 children mothers who had opportunity to breastfeed at work place and mothers who were unemployed/self-employed [12] local practices and existing myths, Lack of support for breastfeeding and Lack of commitment and resources [13]

In an effort to improve optimal breastfeeding practices the Government of Ethiopia gave due emphasis on different initiatives that were developed to reduce under nutrition. For instance the National Nutrition Strategy [14] and the National Nutrition Programmes 2016–2020 [15], and the Seqota Declaration (2015–2030) [16]. Also optimal breast feeding practice was integrated in the Health Sector Transformation Plan (HSTP) [17]

Despite overwhelming evidence of the benefits of optimal breast feeding no study was conducted in this study area therefore this study was aimed at assessing the prevalence of optimal breast feeding practice and associated factors among mothers who had children aged less than or equal to two years.

## Materials and methods

### Study design and setting

A community-based analytical cross-sectional study was conducted from April 15^th^–25^th^, 2018 in Worabe town, which is the capital city of Siltie. Siltʼe is a Zone in the Ethiopian Southern Nations, Nationalities and Peoples' Region (SNNPR) which is located at the 172km away from Addis Ababa capital city of Ethiopia to south west direction and 107km from Hawassa capital city of SNNPR. According to the 2007 National Housing and Population Census, the projected population of Worabe town for the year 2014/15 was about 15,920 and the estimated number of households was 3249. According to the town administration report the town has Six Kebeles (smallest administrative unit of Ethiopia) [18].

### Sample size and sampling procedure

A total of 347 mothers were sampled by using a single population proportion formula with assumption of p 41.4% from study conducted in Bahir Dar, Ethiopia of individuals knowledgeable about breastfeeding [19], d being the expected margin of error (5%), and Z = standard normal distribution with confidence interval of 95% = 1.96.

The obtained sample size was allocated proportionally to the number of households in each Kebeles. To reach the study unit systematic sampling technique was applied with sampling interval of 22 which determined by dividing the total number of households (7610) who have children greater than or equal to two years in the town by calculated sample size. The first house was selected by using pinpoint method and data were collected from household in every 22^nd^ interval.

### Inclusion and exclusion criteria

Mother who had children greater than or equal to two years were included in the study while mothers who were unable to communicate due to serious illness at the time of data collection, those who are unable to speak and hear and mothers who had preterm children were excluded.

### Data collection tool and procedure

Data collection tool was adopted from WHO/UNICEF global strategy on infant and young child feeding practices [1]. Data was collected by a face-to-face interview using a pre-tested, structured questionnaire. Mothers were asked to provide information about their socioeconomic characteristics, education, optimal breastfeeding practices, and obstetric characteristics.

Mothers having child greater than or equal to two years old were asked optimal breastfeeding practice questions (initiation of breastfeeding within one hour of delivery, giving colostrum, exclusive breastfeeding for 6 months, introducing complementary food at 6 months and continued breastfeeding up to 2 years). Those mothers who correctly scored above mean to optimal breastfeeding practice questions were considered as having a good practice.

### Study variable

Socio-demographic characteristics (religion, ethnicity, marital status, education level, household income, family size, age, and occupational status), obstetric factors (e.g. number of pregnancies, frequency of antenatal care visits, postnatal follow-ups, and postnatal care visits), contextual factors (access to information about breastfeeding, place of delivery, and mode of delivery), infant factors (sex), and knowledge (assessed by 5 items; the mean score for knowledge is 2.5 and respondents those who scored greater than or equal to 2.5 were considered as having good knowledge, while those who scored below 2.5 were considered as having poor knowledge (20).

### Data quality control

The data collection tool was translated into a local language, Siltigna, and the collected data were translated back to English by a proficient translator to ensure consistency and accuracy. Training was given to data collectors and supervisors on data collection tool. We pretested the questionnaire on 5% of the calculated sample size out of the study area.

### Data analysis

The data were first coded, entered and cleaned using EpiData statistical software version 3.1 and then exported into SPSS statistical software version 22 for analysis. Descriptive statistics were computed to determine frequencies and summary statistics. Variables with P-value <0.25 in bivariate analysis were selected as a candidate for multi variable logistic regression. Hosmer Lemshow and Omnibus tests were done to test for model fitness. Multi-collinearity was also checked to see the linear correlation among the independent variables by using variance inflation factor and standard error. Variables with variance inflation factor >10 and standard error of > 2 were dropped from the multivariable analysis Finally, P value < 0.05, at 95% confidence interval was declared as statistically significant.

### Ethical clearance

Ethical approval was first obtained from the Ethical Clearance Committee of Wolaita Sodo University prior to data collection. Official letters of co-operation were written to all concerned bodies to obtain their co-operation in facilitating the study. Data collectors obtained informed verbal and written consent form individual participants about the purpose and benefit of the study along with their right to refuse the participation. For participants under 16 years old informed consent was obtained from their parent or guardian.

## Result

### Socio demographic characteristics

The response rate was 92.5%. One hundred and ninety-six (61.0%) were in the age group 25–34 years. One hundred seventy one (53.2%) of the respondents index child were females. Two hundred twenty-five (70.0%) of the participants were Silte and 247 (76.9%) were Muslim. Two hundred and fifty-eight (80.4%) were married and 158 (49.2%) were housewives. One hundred and thirty- nine (43.3%) of respondents had a monthly household income of less than 1500 ETB. Among the respondents 109 (42.2%) of their Husbands were merchant in occupation and 103 (39.9%) were attended more than secondary school. Two hundred and twenty-five (70.0%) of respondents has a family size of less than 4 (Table 1).

**Table 1:** Socio-demographic characteristics of respondents, Worabe town, Siltie zone, 2018 (n = 321).

Characteristics	Category	Frequency(N)	Percent (%)
**Age of mother (Years)**	15–24	54	16.8
	25–34	196	61.1
	35–49	71	22.1
**Sex of last child**	Male	150	46.7
	Female	171	53.3
**Religion**	Muslim	247	76.9
	Orthodox	48	15.0
	Others ^1^	26	8.1
**Ethnicity**	Silte	225	70.1
	Gurage	54	16.8
	Other^2^	42	13.1
**Marital status**	Married	258	80.4
	Single	48	15.0
	Others^3^	15	4.7
**Educational status of mothers**	No formal education	34	10.6
	Primary school	131	40.8
	Secondary school	87	27.1
	More than secondary	69	21.5
**Mother’s occupation**	House wives	158	49.2
	Government employee	81	25.2
	Other^4^	82	25.5
**Husband education (n = 258)**	No formal education	26	10.0
	Primary school	50	19.3
	Secondary school	103	40.0
	More than secondary	79	30.6
**Husband occupation (n = 258)**	Government employee	91	35.3
	Merchant	109	42.2
	Private employee	38	14.7
	Daily laborer	20	7.8
**Total household Income per month in ETB**	<1500**	139	43.3
	1500–3000**	68	21.2
	3000–5000**	35	10.9
	>5000**	79	24.6
**Family size**	<4	225	70.1
	> = 4	96	29.9

Others^1^ Protestant and catholic

Others^2^ Oromo Wolaita

Others^3^ Widowed and Divorced

Others^4^ merchant, private employee and daily labourer

** Ethiopian Birr

### Obstetric characteristics

More than half of mothers 214 (66.7%) had given birth to over three children. Two hundred and six (64.2%) of the respondents had Antenatal care follow-up for the last pregnancy and out of whom 140 (43.6%) had four Antenatal care visits. One hundred and ninety-one (59.5%) delivered in a health facility. Among those delivered at a health facility, 142 (44.2%) and 92 (28.6%) mothers were counselled about breast feeding after delivery and Post-natal care follow-up visit respectively (Table 2).

**Table 2:** Distribution of study subjects by obstetric characteristics of, Worabe town, Siltie zone, 2018 (n = 321).

Variable	Frequency (N)	Percent (%)
**Number of pregnancies**		
<3	107	33.3
≥3	214	66.7
**Antenatal care follow up visit**		
Yes	206	64.2
No	115	35.8
**Number of antenatal care follow up visit of last child**		
1	84	26.2
2	33	10.3
3	64	19.9
4	140	43.6
**Place of delivery**		
Home	130	40.5
Government health institution	144	44.9
Private institutions	47	14.6
**Mode of delivery**		
Spontaneous (normal)	256	79.8
Caesarean section	65	20.2
**Counselled about breastfeeding during last delivery**		
Yes	142	44.2
No	179	55.8
**Postnatal care follow-up visit following last delivery**		
Yes	118	36.8
No	203	63.2
**Counsel about breastfeeding during postnatal care follow up**		
Yes	92	28.7
No	229	71.3

### Knowledge of optimal breastfeeding

**Fig 1:**
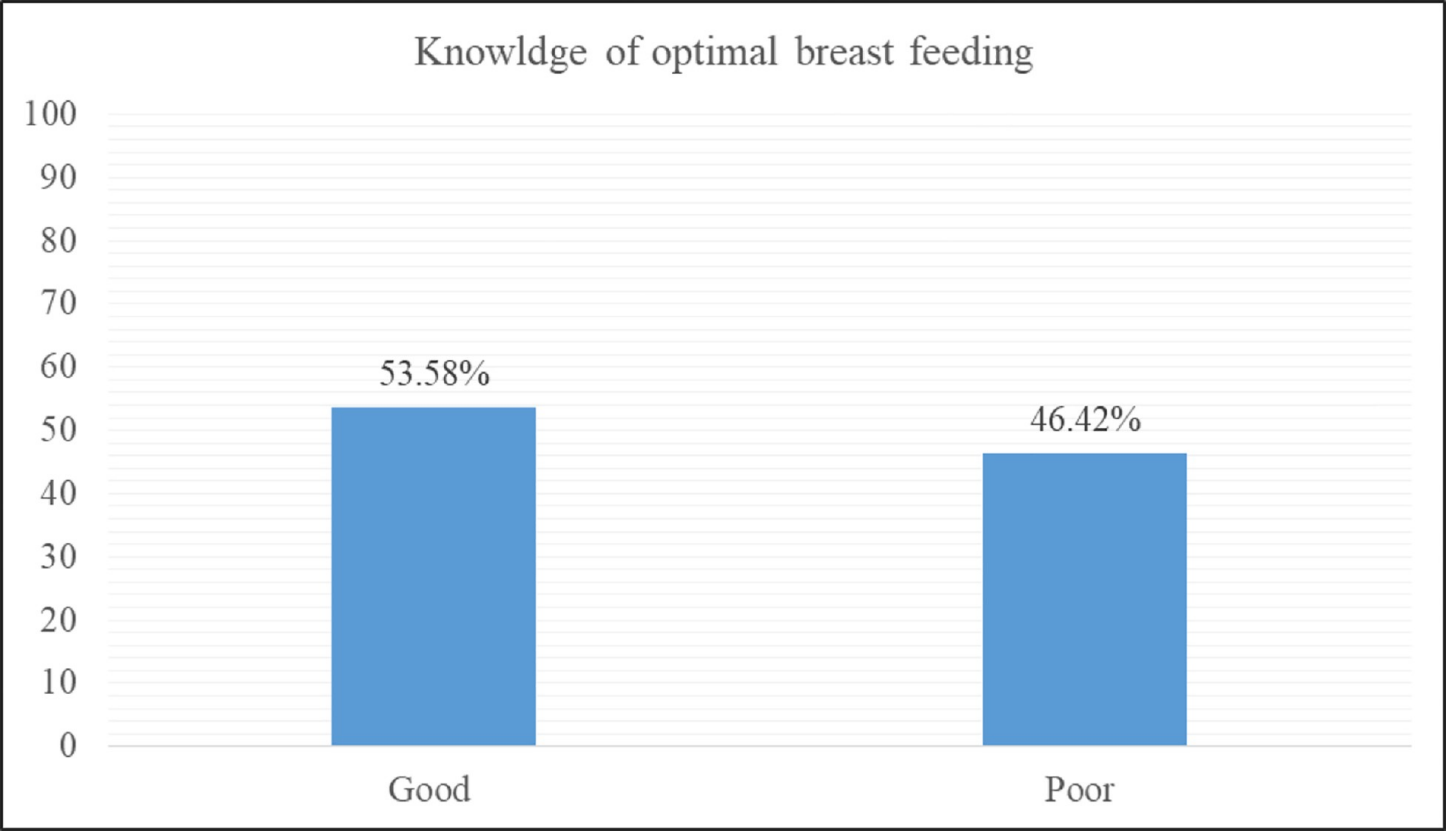
Knowledge of study subjects on optimal breastfeeding, Worabe town, Siltie zone (n = 321).

One hundred seventy two (53.58%) had good knowledge about breast feeding while 149 (46.42%) had poor knowledge (Fig 1).

### Optimal breast feeding practices

**Fig 2:**
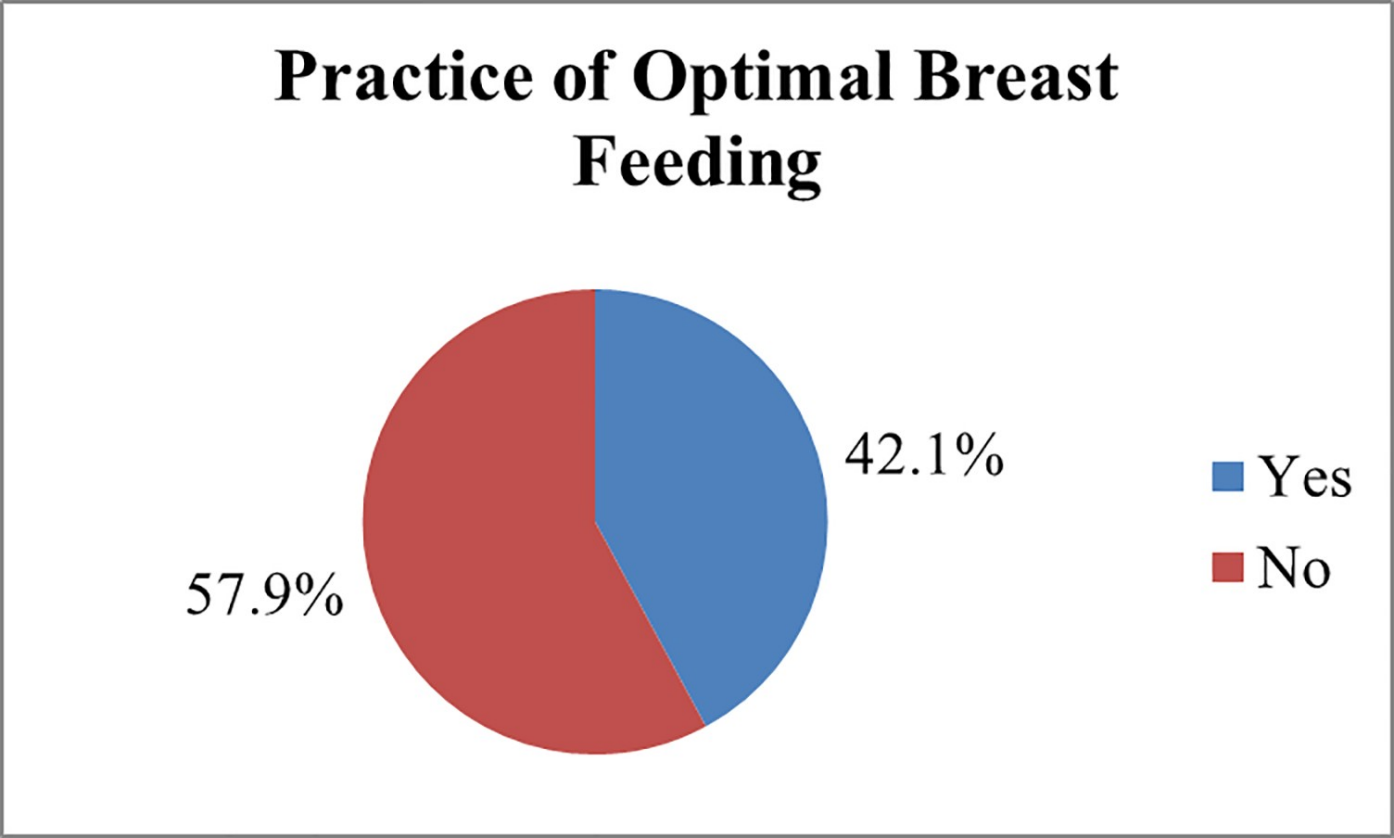
Optimal breastfeeding practice of the study subjects, Worabe town, Siltie zone (n = 321).

One hundred and thirty-five (42.1%) mother’s practiced optimal breast feeding (Fig 2).

### Associated factors with optimal breast feeding practice

Fifteen variables in binary logistic regression with p value of ≤ 0.25 and became a candidate for multiple logistic regressions (Table 3).

**Table 3:** Bivariate and multivariate analysis of socio demographic, knowledge and obstetric related factors with optimal breast feeding practice 2018(n = 321).

Variable	Category	OBF practice		COR,95%CI	P value	AOR,95%CI	P value
		Yes	No				
Age of mother	15–24	15(27.8%)	39(72.2%)	0.49(0.19,0.89)	**0.024**	0.59(0.07, 4.61)	0.620
	25–34	86(43.9%)	110(56.1%)	0.85(0.49, 1.46)	0.561	1.12(0.25, 4.96)	0.872
	>35	34(47.9%)	37(52.1%)	1		1	
Marital status	Married	114(44.2%)	144(55.8%)	1		1	
	Single	13(27.1%)	35(72.9%)	0.46 (0.23,0.92)	**0.030**	0.36(0.04, 2.76)	0.331
	Others^2^	8(53.3%)	7(46.7%)	1.44(0.50,4.09)	0.491	5.25(0.20, 132.36)	0.313
Mothers educational	No formal education	9(26.5%)	25(73.5%)	0.41(0.17, 1.02)	**0.055**	0.19(0.02, 1.64)	0.132
	Primary school	60(45.8%)	71(54.2%)	0.97(0.54, 1.75)	0.938	1.78 (0.33, 9.66)	0.502
	Secondary school	34(39.1%)	53(60.9%)	0.74(0.39, 1.40)	0.360	0.95 (0.17, 5.24)	0.953
	More than secondary	32(46.4%)	37(53.6%)	1		1	
Mothers occupation	House wives	60(38.0%)	98(62.0%)	1		1	
	Gov’t employee	36(44.4%)	45(55.6%)	0.76(0.75, 2.25)	0.335	8.05(1.77, 36.43)	0.007*
	other	39(47.6%)	43(52.4%)	0.88(0.86, 2.54)	**0.153**	8.34(2.02, 34.44)**	0.003*
Total HH income per month in ETB	<1500	56(40.3%)	83 (59.7%)	1		1	
	1500–3000	34(50.0%)	34(50.0%)	1.48(0.82, 2.65)	**0.187**	4.62(1.06, 20.11) *	0.041*
	3000–5000	15(42.9%)	20(57.1%)	1.11(0.52, 2.35)	0.782	6.31(0.86, 46.00)	0.069
	>5000	30(38.0%)	49(62.0%)	0.90(0.51, 1.60)	0.737	2.27 (0.50, 10.13)	0.282
Family size	<4	88(39.1%)	137(60.9%)	1		1	
	> = 4	47(49.0%)	49(51.0%)	1.49(0.92, 2.41)	**0.103**	0.79(0.20, 3.10)	0.742
Knowledge optimal breast feeding	Good	66(38.4%)	106(61.6%)	0.72(0.46, 1.12)	**0.151**	5.51 (1.67,18.14)*	0.005*
	Poor	69(46.3%)	80(53.7%)	1		1	
No. of pregnancy	<3	39(36.4%)	68(63.6%)	0.70(0.43, 1.13)	**0.151**	0.58(0.15, 2.12)	0.412
	≥3	96(44.9%)	118(55.1%)	1		1	
ANC follow up visit	Yes	93(45.1%)	113(54.9%)	1		1	
	No	42(36.5%)	73(63.5%)	1.43(0.89, 2.28)	**0.134**	0.15 (0.04,0.57)*	0.005*
No. of ANC follow up visit of last child	1	40(47.6%)	44(52.4%)	1.24(0.72, 2.15)	0.425	2.03(0.52, 7.89)	0.305
	2	17(51.5%)	16(48.5%)	1.45(0.68, 3.12)	0.331	1.81(0.33, 9.70)	0.489
	3	19(29.7%)	45(70.3%)	0.58(0.30, 1.09)	**0.091**	0.32(0.06, 1.77)	0.196
	4	59(42.1%)	81(57.9%)	1		1	
Place of delivery	Home	61(46.9%)	69 (53.1%)	1		1	
	Gov’t institution	52(36.1%)	92(63.9%)	0.63(0.39, 1.03)	**0.070**	0.56(0.17, 1.78)	0.328
	Private institution	22(46.8%)	25(53.2%)	0.99(0.51, 1.94)	0.989	1.51 (0.26, 8.56)	0.639
Sex of last child	Male	69(46.0%)	81(54.0%)	1.37(0.88, 2.19)	**0.180**	0.62(0.21, 1.77)	0.373
	Female	66 (38.6%)	105(61.4%)	1		1	
Mode of delivery	Spontaneous	99(38.7%)	157(61.3%)	1		1	
	Cesarean section	36(55.4%)	29(44.6%)	1.96(1.13, 3.41)	0.016	4.23(1.27, 14.13)*	0.019*
PNC follow-up visit of last delivery	Yes	43(36.4%)	75(63.6%)	1.44(0.90, 2.30)	0.121	1.45(0.20, 10.19)	0.709
	No	92(45.3%)	111(54.7%)	1		1	
Counseled on breastfeeding during PNC follow up	Yes	33(35.9%)	59(64.1%)	0.44 (0.87, 2.36)	0.156	4.94(1.31, 10.19)*	0.018*
	No	127(55.5%)	102(44.5%)	1		1	

* = p-value <0.05, CI = Confidence Interval, COR = Crude Odds Ratio, AOR = Adjusted Odds Ratio PNC = Post Natal Care

In the multivariate logistic regression; Knowledge of optimal breast feeding, total household income per month, Woman's occupation, breastfeeding counselling during postnatal care follow up, having antenatal care follow up and mode of delivery, were significantly associated (p<0.05) with optimal breast feeding practice. But (Table 3)

Government employee and other mothers (merchant, private employee and daily labourer) were 8.0 and 8.3 times more likely to practice optimal breast feeding (AOR = 8.0; 95% CI 1.7 to 36.4) and (AOR = 8.3; 95% CI 2.0 to 34.4) than house wives, respectively.

Family’s whose household income 1500–3000 Ethiopian birr were 4.6 times more likely to practice optimal breast feeding than whose household income less than 1500 Ethiopian birr (AOR = 4.6; 95% CI 1.0 to 20.1)

Mothers who had a good knowledge of optimal breast feeding were 5.5 times more likely to practice optimal breast feeding than that of poor knowledge (AOR = 5.5; 95% CI 1.6 to 18.1)

Mothers who counselled about breast feeding during post-natal follow up were 5 times more likely to practice optimal breast feeding than their counterpart (AOR = 4.9; 95% CI 1.3 to 10.1). Mothers who have no antenatal care follow-up visit 84.1% less likely to practice optimal breast feeding than their counterparts (AOR = 0.1; 95% CI 0.04 to 0.5).

Mothers who delivered by caesarean section 4.2 times more likely to practice optimal breast feeding than those delivered spontaneously.(AOR = 4.2; 95% CI 1.2 to 14.1) (Table 3).

## Discussion

This study has provided vigorous information on Predictors of optimal breast feeding practice up to two years and factors associated with practice in Worabe town. The finding of this study showed that having knowledge of optimal breast feeding, total household income per month, mothers occupation, breastfeeding counselling during postnatal care follow up, having antenatal care visit and mode of delivery, were factors affecting of optimal breast feeding practice.

The overall practice of optimal breast feeding in this study was 42.1% which is higher than study carried out in Jimma Arjo Wereda 24.6% [20], Bishoftu (8.8%) [21], Hula district [22] and Gonder town [23]. This might be due to low accessibility of commercial food and living in less urbanized area has an effect on choice of infant feeding. And also most participants in this study are house wives; this may have an impact for the high prevalence.

However, it is significantly lower than the finding from Tanzania (51.1%) [24], this might be variation in sample size and socio-demographic characteristics on our study.

It was observed that government employed and others mothers (merchant, private employee and daily labourer) were 8.0 and 8.3 times more likely to practice optimal breast feeding (AOR = 8.0; 95% CI 1.7 to 36.4) and (AOR = 8.3; 95% CI 2.0 to 34.4) than house wives respectively. This finding is inconsistent with studies conducted in Bishoftu [21]. The possible explanation might be during this study period the national maternal leave was extended to four months and additional two month annual leave opportunities was created in Ethiopia which might increase the practice of government employee.

Family’s whose household income 1500–3000 Ethiopian birr were 4.6 times more likely to practice optimal breast feeding than whose household income less than 1500 Ethiopian birr (AOR = 4.6; 95% CI 1.0 to 20.1). This finding is inconsistent with finding in Bishoftu [21] and Masha district Hadya zone [10]. This could be, those family with high income can afford their household expenses easily and able to consume balanced diet for the mother and don’t fear maternal malnutrition while breast feeding their child and also they have more access for different technology to gather information on breast feeding.

Mothers’s who has good knowledge of optimal breast feeding were 5.5 times more likely to practice optimal breast feeding than that of poor knowledge (AOR = 5.5 95% CI 1.6 to 18.1). This is in line with a finding from Pakistan city[25] and Arbaminch Zuriya Woreda [26].This might be due to an in-depth knowledge of optimal breast feeding may increase mother’s understanding on the importance of optimal breast feeding, and thus, increases practicing. But this finding is inconsistent in Lahore Pakistan [27] this might be, some mothers are resistant to apply knowledge to practice.

Mothers who were counselled about breast feeding during post-natal follow up 5 times more likely to practice optimal breast feeding than their counterpart (AOR = 4.9; 95% CI 1.3 to 10.1). This is in line with a finding from Gonder town [23] and Masha district Hadya zone [10]. This might be post-natal period is a critical period for implementation of appropriate infant feeding intervention strategies to promote optimal breastfeeding behaviour and the health care providers are doing their job.

Mothers who has no antenatal care follow up visit 84.1% less likely to practice optimal breast feeding than their counterpart (AOR = 0.1; 95% CI: 0.04, 0.5). This is in line with a finding from Masha district Hadya Zone [25], Nigeria [28] and Croatia [29]. This might be due to the fact that the mothers who has no information before has less likely to practice.

Accordingly mothers who delivered by caesarean section 4.2 times more likely to practice optimal breast feeding than those delivered spontaneously.(AOR = 4.2 95% CI 1.2 to 14.1). This is inconsistent with finding of Gonder town [23]. The possible explanation for this might be related to the increased prevalence of caesarean section in sub-Saharan Africa [30] offers no option to cease breast feeding their child early.

Generally this study generated rich information on optimal breast feeding practice in the community and study was done in all Kebele’s and used probability sampling technique so findings can be generalized the whole country. But the research might have faced the following biases; social desirability bias and recall bias since mothers’ were asked for the past experience before two years.

## Conclusion

The finding of this study showed that prevalence of optimal breast feeding practice was 42.1%. Having knowledge of optimal breast feeding, total household income per month, mothers occupation, breastfeeding counselling during postnatal care follow up, having antenatal care visit and mode of delivery, were factors affecting of optimal breast feeding practice. Awareness creation programs through health education should be strongly done in the communities in collaboration with health extension workers, and zonal health offices to avoid child mortality on this critical period and continuous follow up for its implementation.

## Supporting information

S1 File (DOCX)

S2 File (DOCX)

## Abbreviations

ANC: Antenatal Care
AOR: Adjusted Odds Ratio
COR: Crud Odds Ratio
ETB: Ethiopian Birr
HH: House Hold
OBF: Optimal Breast Feeding
PNC: Post Natal Care
