# Supplementary material for: Predictors of optimal breastfeeding practices in Worabe town, Silte zone, South Ethiopia
Source: PLoS One. 2020 Apr 30;15(4):e0232316. doi: 10.1371/journal.pone.0232316 (PMC7192429; doi:10.1371/journal.pone.0232316)
Supplement: S1 File — (DOCX) [file pone.0232316.s001.docx]

Questionaries’ of optimal breast feeding practice

Part 1 socio-demographic characteristics

| s. no | Questions | Choice | Skip |
| --- | --- | --- | --- |
| 101 | How old are you?( in Complete year) | ____________ |  |
| 102. | What is your ethnicity? | 1. Siltie 2. Gurage 3. Woliata 4. Amhara 5. Other specify |  |
| 103 | What is your religion? | 1. Muslim 2. Orthodox 3. Protestant 4. Catholic 5. Other specify |  |
| 104 | What is your marital status? | 1. Single 2. Married 3. Widowed 4. Divorced |  |
| 105 | What is your highest level of education? | 1. No formal education 2. Primary (1-8) school 3. Secondary (9-12) school 4. More than secondary |  |
| 106 | What is your occupation? | 1. Government employee 2. Private employee 3. Merchant 4. Daily labor |  |
| 107 | What is your husband highest level of education? | 1. No formal education 2. Primary (1-8) school 3. Secondary (9-12) school 4. More than secondary |  |
| 108 | What is your husband occupation? | 1. Government employee 2. Private employee 3. Merchant 4. Daily labor |  |
| 109 | What is your average family income in Ethiopian birr? | ________________ |  |

Part 2 obstetrics characteristics

| s.no | Question | Choice | Skip |
| --- | --- | --- | --- |
| 201 | How many pregnancies do you have ? | _________ |  |
| 202 | Did you have ANC care follow-up visits in any health facility while you were pregnant for the last child? | 1. Yes 2. No |  |
| 203 | How many ANC follow-up visits did you attend? | 1. ___ 2. ___ 3. ____ 4. And above |  |
| 204 | What is the sex of your last child? | 1. Male 2. Female |  |
| 205 | How old is your last child? (in months) | _______ |  |
| 206 | What kind of delivery? | 1. Normal 2. Cesarean |  |
| 207 | Where did you give birth to your last child? | 1. Home 2. Government health institution 3. Private institutions |  |
| 208 | Have you get counseled/ advised about breast feeding during your last delivery? | 1. Yes 2. No |  |
| 209 | Did you have PNC follow-up visit during last delivery? | 1. Yes 2. No |  |
| 210 | Do you got counseling about breast feeding in your PNC follow-up visits? | 1. Yes 2. No |  |

Part 3 knowledge related questions

| s.no | Question | Choice | Skip |
| --- | --- | --- | --- |
| 301 | Initiation of breastfeeding after birth of last child | 1. Immediately within one hour 2. After one hour |  |
| 302 | Is any food/drink given before six month | 1. Yes 2. No |  |
| 302 | Is first milk (colostrum) important for child? | 1. Yes 2. No |  |
| 303 | For how many months should a child feed only breast milk? | 1. < 6 months 2. ≥ 6 months |  |
| 304 | How many months should a child feed breast milk? | 1. < 24 month 2. ≥ 24 month |  |

Part 4 practice related questions

| s.no | Question | Choice | Skip |
| --- | --- | --- | --- |
| 301 | When did you started breastfeeding after birth of your last child? | 1. Immediately within one hour 2. After one hour 3. After 24 hour |  |
| 302 | Have you given food/drink given before six month for your child? | 1. Yes 2. No |  |
| 302 | Is first milk (colostrum) important for child? | 1. Yes 2. No |  |
| 303 | For how many months did you fed your last child only breast milk? | 1. < 6 months 2. ≥ 6 months |  |
| 304 | For how many months did you fed breast milk your last child? | 1. < 24 month 2. ≥ 24 month |  |
