# Supplementary material for: Predictors of optimal breastfeeding practices in Worabe town, Silte zone, South Ethiopia
Source: PLoS One. 2020 Apr 30;15(4):e0232316. doi: 10.1371/journal.pone.0232316 (PMC7192429; doi:10.1371/journal.pone.0232316)
Supplement: S2 File — (DOCX) [file pone.0232316.s002.docx]

ላጦቢሎ ያስናጄ ሱል

1. የአመሰብ ሃላት

| ኢልቅ | ሱል | ሚጥሮት | አቲልፍ |
| --- | --- | --- | --- |
| 101 | ኡምራሽ ሚስትን?( በዘማን) | ____________ |  |
| 102. | ጊቻሽ ምንግዝን? | 1. ስልጤ 2. ጉራጌ 3. ወላይታ 4. አማረ 5. ገናም ቦነ ኢዉድ |  |
| 103 | ቲትኬተይያሺ ዲን ምንግዝን? | 1. ሙስሊም 2. ኦርቶዶክስ 3. ጴንጤ 4. ካቶሊክ 5. ገናም ቦነ ኢዉድ |  |
| 104 | የጎጃሽ ሃለት ? | 1. አላገባሆ 2. አገባሆ 3. ሞታን 4. ተላዬናን |  |
| 105 | ጎትተኜይ የአሽር መቃማሽ ምስትን? | 1. አልቀራሆ 2. ሀድለኜ መቃም (1-8) አሽር ጋር 3. ሆሽትለኜ መቃም (9-12) አሽር ጋር 4. ቶሽትለኜ መቃም ደር |  |
| 106 | ቢላሽ ምንግዝን? | 1. የመንግስት ብለተኘ 2. የቢቶ ብለተኘ 3. ዘልዛሎ 4. ያያም ብለተኜ |  |
| 107 | የጋራሽ አቦት አሽረ ምስተ ጃንጎ ቀራን ? | 1. አሽር አልቀራን 2. ሀድለኜ መቃም (1-8) አሽር ጋር 3. ሆሽትለኜ መቃም (9-12) አሽር ጋር 4. ቶሽትለኜ መቃም ደር |  |
| 108 | የጋራሽ አቦት ብልከ ምንግዝን? | 1. የመንግስት ብለተኘ 2. የቢቶ ብለተኘ 3. ዘልዛሎ 4. ያያም ብለተኜ |  |
| 109 | ያበሮሳሽ ጉትለኜ ገቢከ ቦሪ በቶጰ ቢር ምስትን? | ________________ |  |

1. የጪኖት ሃላት

| ኢልቅ | ሱል | ሚጥሮት | አቲልፍ |
| --- | --- | --- | --- |
| 201 | ሚስተጊን ሆሸተፎል ሆንሼሽ? | _________ |  |
| 202 | ለቁጢሶይ ወልዳሽ ተጬኚ ቀዳ በአፊያ ጋር ለፈይነትከ ተክታተልሽኒያሽ ወይ? | 1. አዎ 2. አልቲክታልኮ |  |
| 203 | ሚስተ ጊን ሄድሼሽ? | 1. ___ 2. ___ 3. ____ 4. ታራት ጊን ደር |  |
| 204 | ቁጢሶይ ወልዳሽ ምንግዝን? | 1. ልጅን 2. ገረድን |  |
| 205 | ቁጢሶይ ወልዳሽ ኡምሪክ ቦሪ ሚስትን? | _______ |  |
| 206 | የጬኜሽቢ ሃለት አይነኮን? | 1. በፈያን 2. በስደቆት |  |
| 207 | ቁጢሶይ ወልዳሽ ባኤን ጬኜሼሽ? | 1. በጋር 2. በመንግስት አፊያ ጋር 3. በግሊ አፊያ ጋር |  |
| 208 | ቁጢሶ በጬሺ ወልዳሽ አይነኮ ታጦቢያሽኮ ሸዣ ኤወዱሻን ወይ? | 1. ኣዎ 2. አሌወደኛን |  |
| 209 | ቁጢሶ በጬሺ ወልዳሽ ለፈይነትከ አፊያ ጋር ተክታታልሽኒያሽ ወይ ? | 1. አዎ 2. አልቲክታተልኮ |  |
| 210 | በቁጢሶይ ወልዳሽ ለአፊያከ ቲትክታታይ አይነኮ ታጦቢያሽኮ ኤወዱሻን ወይ? | 1. አዎ 2. አሌወዱኛን |  |

1. የእልም ቻሎት ተምሳሳዬ ሱል

| ኢልቅ | ሱል | ሚጥሮት | አቲልፍ |
| --- | --- | --- | --- |
| 301 | ቁጢሶይ ወልዳሽ ተጬሺኮ አጥቦት በምስት ሳት ጀመርሽኒ? | 1. ባድ ሳት ጉት 2. ታድ ሳት ዞፍ |  |
| 302 | ቁጢሶይ ወልዳሽ በጬኜ ዞፍ ተስድስት ወሪ ቀዳ ስንቀ ወሰዳን ወይ? | 1. አዎ 2. አሎሰዳን |  |
| 302 | ያፍቴይ የጥብ ሀይብ ሎልድ ፈያን ወይ? | 1. አዎ 2. ኡንኮ ፈያ |  |
| 303 | ወልድ ጡበ ቢቾ ኢጦባነይ ለሚስትን ወቅት? | 1. ተስድሰት ወሪ ኮሎ 2. ስድሰት ወሪዋ ደር |  |
| 304 | ሚስተን ወሪ ወልድ ጡበ ጡቦት ያለቢ? | 1. ተኩያ አራት ወሪ ኮሎ 2. ኩያ አራት ወሪዋ ደር |  |

4 የትራሮሻት ተምሳሳዬ ሱል

| ኢልቅ | ሱል | ሚጥሮት | አቲልፍ |
| --- | --- | --- | --- |
| 301 | ቁጢሶይ ወልዳሽ ተጬሺኮ አጥቦት በምስት ሳት ጀመርሽኒ ናረ? | 1. ባድን ሳት ጉት 2. ታድን ሳት ዞፍ 3. ተኩያ አራትን ሳት ዞፍ |  |
| 302 | ቁጢሶይ ወልዳሽ በጬኜ ዞፍ ተስድስት ወሪ ቀዳ ስንቀ ወሰዳን ናር ወይ? | 1. አዎ 2. አሎሰዳን |  |
| 302 | ያፍቴይ የጥብ ሀይብ ሎልድ ፈያን ናር ወይ? | 1. አዎ 2. ኡንኮ ፈያ |  |
| 303 | ወልድ ጡበ ቢቾ የጦቦዬይ ለሚስትን ወቅት ናረ? | 1. ተስድሰትን ወሪ ኮሎ 2. ስድሰት ወሪዋ ደርን |  |
| 304 | ሚስተን ወሬ ወልዳሽ ጡበ የጦቤይ ናረይ? | 1. ተኩያ አራት ወሪ ኮሎን 2. ኩያ አራት ወሪዋ ደርን |  |
